# Cellular High-Energy Cavitation Trauma – Description of a Novel *In Vitro* Trauma Model in Three Different Cell Types

**DOI:** 10.3389/fneur.2016.00010

**Published:** 2016-02-01

**Authors:** Yuli Cao, Mårten Risling, Elisabeth Malm, Anders Sondén, Magnus Frödin Bolling, Mattias K. Sköld

**Affiliations:** ^1^Department of Neuroscience, Karolinska Institutet, Stockholm, Sweden; ^2^Section of Surgery, Department of Clinical Science and Education, Karolinska Institutet at Södersjukhuset, Stockholm, Sweden; ^3^Department of Oncology-Pathology, Karolinska Institutet, Stockholm, Sweden; ^4^Department of Neurosurgery, Uppsala University Hospital, Uppsala, Sweden

**Keywords:** *in vitro* high-energy cavitation trauma model, flyer-plate, automatic time-lapse imaging, neuroblastoma, glioma, post trauma mechanisms, mitosis, regulated differential gene expression

## Abstract

The mechanisms involved in traumatic brain injury have yet to be fully characterized. One mechanism that, especially in high-energy trauma, could be of importance is cavitation. Cavitation can be described as a process of vaporization, bubble generation, and bubble implosion as a result of a decrease and subsequent increase in pressure. Cavitation as an injury mechanism is difficult to visualize and model due to its short duration and limited spatial distribution. One strategy to analyze the cellular response of cavitation is to employ suitable *in vitro* models. The flyer-plate model is an *in vitro* high-energy trauma model that includes cavitation as a trauma mechanism. A copper fragment is accelerated by means of a laser, hits the bottom of a cell culture well causing cavitation, and shock waves inside the well and cell medium. We have found the flyer-plate model to be efficient, reproducible, and easy to control. In this study, we have used the model to analyze the cellular response to microcavitation in SH-SY5Y neuroblastoma, Caco-2, and C6 glioma cell lines. Mitotic activity in neuroblastoma and glioma was investigated with BrdU staining, and cell numbers were calculated using automated time-lapse imaging. We found variations between cell types and between different zones surrounding the lesion with these methods. It was also shown that the injured cell cultures released S-100B in a dose-dependent manner. Using gene expression microarray, a number of gene families of potential interest were found to be strongly, but differently regulated in neuroblastoma and glioma at 24 h post trauma. The data from the gene expression arrays may be used to identify new candidates for biomarkers in cavitation trauma. We conclude that our model is useful for studies of trauma *in vitro* and that it could be applied in future treatment studies.

## Introduction

Traumatic brain injury (TBI) is a leading cause of disability in people under 40 (1). An estimated 1.5 million die and 150–200 per million TBI cases become severely disabled each year (1, 2). Because the distribution of physical forces that can cause injury in the central nervous system (CNS) is complex, TBI can be focal and/or diffuse (3). For example, focal injuries could result from penetrating objects or focal strikes to the skull. Diffuse injuries could be caused by acceleration/deceleration or blast-TBI (shock waves from detonations), for which there are a number of TBI models (3, 4). Not only the forces are complex but also the different aspects of the head, like the skull, the cerebrospinal fluid (CSF), the different brain regions, and the vasculature with their varied densities, viscosities, and topologies (5–9) add to the complexity of understanding TBI. In order to model these injuries, the complex physics and biology of the head must be taken into account. *In vitro* models could serve as important tools in the study of these different and interacting complex aspects of injury and ultimately the different forces involved in TBI could be studied one at a time.

Though not well understood, cavitation could be one TBI mechanism (10–13). Cavitation usually occurs in liquids subjected to rapid pressure changes from detonation shock waves. A shock wave’s pressure-vs.-time profile has been characterized *in vitro* and *in situ*: a high pressure peak followed by a negative pressure (5, 14–17). In liquids exposed to such drastic pressure change, cavitation bubbles may develop and collapse. Researchers have not been able to observe cavitation in the living brain due to the transient nature of cavitation and the challenge in defining areas where cavitation occurs. *In vitro* models could thus be useful to study the biological effects of cavitation and shock waves.

Primary cultures of endothelial cells have previously been used to investigate shock wave cavitation trauma (SWCT) (14, 18). However, cell lines provide advantages, such as stable biological patterns, high growth rate, and high experimental throughput in comparison to primary cultures, and are therefore frequently used in pharmaceutical efficacy/toxicological studies and screening. In this study, we chose neuroblastoma (SH-SY5Y), glioma (C6), and Caco-2 cell lines that share various properties with neurons, glia, and endothelial cells of the nervous system, respectively, and that we, therefore, found suitable for study of *in vitro* TBI. Tight junctions that make up the blood–brain barrier (BBB) are, for instance, present in capillaries’ endothelial cells and Caco-2, respectively. Cell monolayers were exposed to controlled trauma, the severity of which was controlled, measured, and could be varied. Our aim is to establish a stable *in vitro* system to induce reproducible SWCT on cells. Stability, high throughput, and reproducibility are desired features of a model, which could be obtained by using cell lines and having control over trauma severity. The model will be used to study posttrauma cell biology and treatments.

## Materials and Methods

### Overall Study Design

Monolayers of neuroblastoma (SH-SY5Y), glioma (C6), and Caco-2 were traumatized using the flyer-plate model. Top and bottom cavitations were expected in wells with 400 or 600 μl medium (14, 18). Bottom cavitation alone was expected in wells with 1000 μl medium. Wells with 400 and 1000 μl medium were used to investigate cavitation’s effect on cell monolayers.

The three aspects of cellular responses to shock wave and cavitation that were studied included growth, trauma biomarker expression, and overall gene regulation.

Growth was studied by looking at lesion size and morphological changes, mitosis frequency, and cell counts gained from time-lapse images of the monolayer.

All these experiments were performed on different cell cultures. For each cell line, different cell cultures were used for each experiment (assessment) and no cell cultures for a given cell line were used for multiple experiments.

### Cell Culture and Trauma Model

#### Cell Culture

Rat glioma (C6) was cultured in Ham’s F12 medium supplemented with 15% horse serum and 2.5% fetal bovine serum. Human Caco-2 was cultured in Eagle’s minimum essential medium with 20% fetal bovine serum. Human neuroblastoma (SH-SY5Y) was grown in Dulbecco’s modified Eagle medium with 10% fetal bovine serum and 1% 200 mM l-glutamine. All cells were maintained in 5% CO_2_ air atmosphere, 37°C. Upon confluence in flasks, cells were subcultured on glass cover slips in 24-well cell culture dishes. For Cell IQ and gene array observations, cells were grown on well bottoms without cover slips. C6, Caco-2, SH-SY5Y, Eagle’s minimum essential medium, Ham’s F12 medium, horse serum, and fetal bovine serum are from ATCC (Manassas, VA, USA). Fetal bovine serum, Dulbecco’s modified Eagle media, trypsin–EDTA, and l-glutamine are from Invitrogen (Stockholm, Sweden). *T*-75 cm^2^ cell culture flasks and polystyrene 24-well cell culture dishes are from Nunc (Roskilde, Denmark). Glass cover slips (∅ 13 mm, thickness = 0.15 mm) are from BergmanLabora (Danderyd, Sweden). All controls/sham cultures were treated likewise but not exposed to *in vitro* trauma.

#### The Flyer-Plate Trauma Model

**Figure 1:**
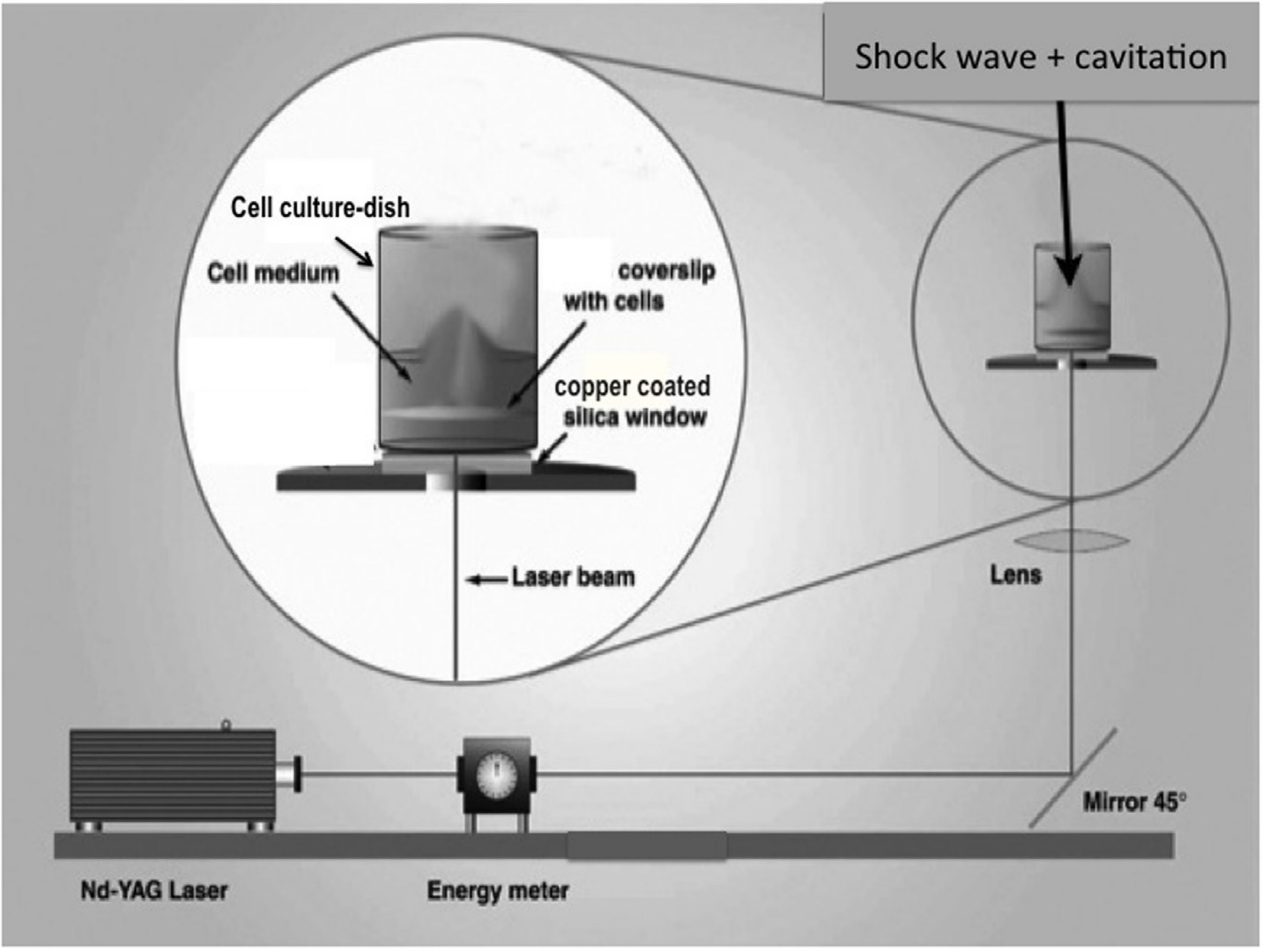
**Schematic of the flyer-plate model, where a cell monolayer is exposed to a shock wave cavitation trauma (SWCT)**. A well is placed atop the copper–silica window, which is hit by a laser. A piece of copper (a flyer-plate) becomes accelerated and hits the well bottom. Cavitation develops at the bottom and the surface of the medium when using 400 or 600 μl medium/well.

A cell monolayer was exposed to a SWCT with the setup illustrated in Figure 1. A pulsed Nd-YAG laser (wavelength 1064 nm) produced laser bursts with energy measuring 500–600 mJ. The laser was directed toward a copper-coated fused silica window. Subjected to the laser, the inner layer of the copper vaporized. Expansion of copper vapors accelerated a piece of the superficial layer of the metal – the flyer-plate. One well at a time (of a 24-well cell culture dish) was placed on top of the copper–silica window, covered by a projector transparency. Without a transparency, the well would become copper stained, against which the transparent cells are not visible and impossible to photograph. The transparency was used in all experiments, except the S100B study. The well bottom was hit by the accelerating flyer-plate. Cavitation developed at the bottom and the surface of the medium when using 400 or 600 μl medium/well. An earlier study has shown that top cavitation always leads to droplet formation on the lid above the well (14). This top cavitation droplet was used as an inclusion criterion for continued observation of the cell culture in question. Without a droplet, the cell culture was not further studied. The model has been described in detail in earlier studies (14, 18). Every exposed colony received one SWCT insult, except those colonies used for the S100B measurements, which received two insults.

A well with 400 μl medium was used for all cell cultures with the following exceptions: 1000 μl medium/well to see whether or not the lesion size will differ with varying medium volumes, 600 μl medium/well [600 μl was necessary for enzyme-linked immune-sorbent assay (ELISA)] for the S100B observations (the cultures were exposed to repeated trauma within 1–3 min of the first trauma). The medium was carefully added to the well to avoid bubbles. Any visible bubbles were removed with a pipette. After exposures, the medium was removed from the cell culture wells, and 1000 μl fresh medium (37°C) was added to each well. Cells were maintained as prior to the trauma. Tables 1–5 show the number and type of unique cultures used for each experiment. For the S100B study, there was no post trauma medium replacement.

**Table 1:** **Number of colonies and cell type used in assessment of lesion size with varied medium volumes**.

	0.4 ml medium/well upon exposure	1 ml medium/well upon exposure
Neuroblastoma	*n* = 13 colonies	*n* = 16 colonies
Glioma	*n* = 32 colonies	*n* = 16 colonies
Caco2	*n* = 24 colonies	*n* = 16 colonies

*The same cultures were photographed at five time intervals: 0.75–1.00, 3.33–4.33, 5.50–6.17, 23.00–23.50, and 26.00–26.33 h post trauma*.

**Table 2:** **Number of observation (*n* = number of unique cultures) in colonies used for calculation of percentage mitosis in lesion periphery, confluent zone, and control**.

	Lesion periphery	Confluent zone	Control	Medium volume (ml)
Neuroblastoma	20 (*n* = 18)	18 (*n* = 18)	17 (*n* = 17)	0.4
Glioma	13 (*n* = 13)	17 (*n* = 13)	19 (*n* = 19)	0.4

**Table 3:** **Number of colonies used in the cell IQ assessment**.

	No. of traumatized colonies	No. of controls	Medium volume (ml)
Neuroblastoma	*n* = 19	*n* = 14	0.4
Glioma	*n* = 20	*n* = 13	0.4
Caco2	*n* = 16	*n* = 8	0.4

**Table 4:** **Number of glioma colonies used in the S100B experiments for each exposure condition**.

	No. of insults/colony	S100B assessed colonies	Medium volume/well upon exposure (ml)
Glioma controls	0 insult	*n* = 6 colonies	0.6
Glioma trauma colony	1 insult	*n* = 6 colonies	0.6
Glioma trauma colony	2 insults	*n* = 8 colonies	0.6

**Table 5:** **Number of traumatized and control cultures used for gene array analysis**.

	No. of colonies in each sample	No. of samples		Medium volume (ml)
Neuroblastoma	1 colony	3 traumatized	3 controls	0.4
Glioma	8 colonies	3 traumatized	3 controls	0.4

The Nd-YAG-laser, Brilliant B model, is from Quantel (Paris, France). The energy meter, model DGX with a high-energy probe model 30-A-P-RP-DIF, is from Ophir (Jerusalem, Israel). The laser beam is redirected with a high-energy laser mirror (type 08MLQ005/426) and focused with a fused silica symmetric convex lens (type 01LQB235), from Melles Griot (Stockholm, Sweden). Fused silica windows (∅ 32 mm, thickness = 1.5 mm) are from Werner Glas AB (Stockholm, Sweden). The silica window is coated with 0.7 μm copper by The Swedish Defense Research Agency (Stockholm, Sweden). For visual guidance, we used a stereomicroscope SMZ-2T from Nikon (Tokyo, Japan), a CCD Iris Color Video Camera SSC-C370P from Sony (Stockholm, Sweden), and a monitor LDH 2106/00 from Philips (Eindhoven, The Netherlands).

### Post Trauma Growth

#### Lesion Size and Morphology

**Figure 2:**
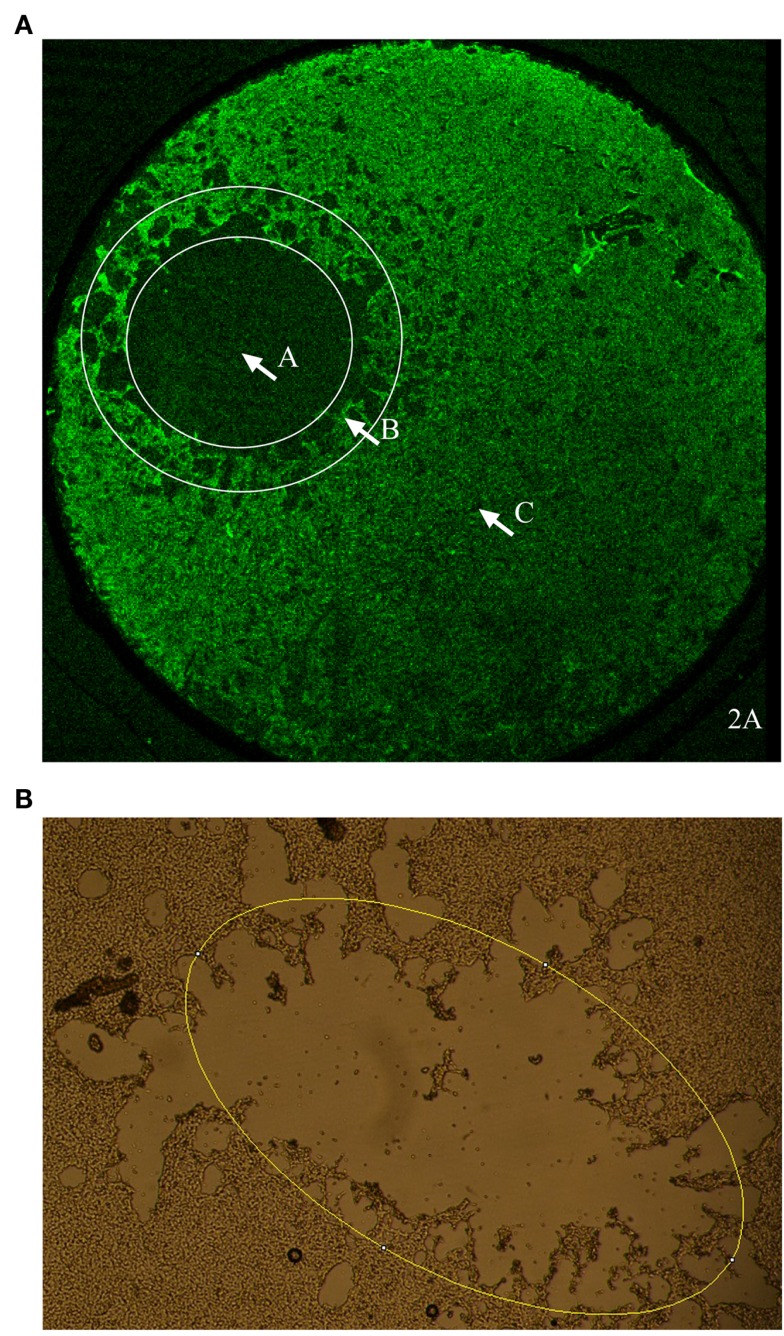
**(A)** An overview of the morphology of a cell culture of neuroblastoma after *in vitro* flyer-plate trauma. Zone A is the central lesion area, devoid of cells. Zone B is the lesion periphery where there are still viable cells mixed with dying cells and cell debris. Zone C is the confluent cell layer with unaffected cells. **(B)** Example of how the elliptical tool was applied. ImageJ’s elliptical tool was used to give an approximation of the lesion size. As shown in the picture every ellipse was drawn to follow the lesion periphery. Lesion periphery was defined as the area outside the central area devoid of cells but with cells in a more scattered pattern than in the confluent zone.

The flyer-plate traumatized cell cultures were observed live using a Nikon diaphot 300 phase contrast microscope (Tokyo, Japan) and a mounted Nikon D3100 digital camera (Tokyo, Japan). The same cell cultures were photographed at five time intervals (Table 1). The lesions were assessed for area and perimeter using the image analysis program, ImageJ64 (NIH). The trauma produces a cell-free area centrally within the injury zone. Outside this area, there is a border zone with a mix of cell debris and viable cells in a scattered pattern (the lesion periphery) followed by a zone of confluent cells (see Figure 2A). Lesion periphery was defined as the area outside of the central area devoid of cells but with cells in a more scattered pattern than in the confluent zone (see Figure 2A). ImageJ’s elliptical tool (Figure 2B) was manually used by the same researcher to give a consistent selection of the lesion on each image. Every ellipse was placed within this lesion periphery (see Figure 2B). Cultures of neuroblastoma are shown in Figures 2A,B.

The measured lesion area and perimeter were normalized in excel: the first time point lesion area/perimeter was defined as 100%, and every lesion area/perimeter thereafter was reported as a percentage of the first value. Statistical analyses, performed using Prism 5 for MacOS X (version 5.0d from GraphPad, La Jolla, CA, USA), were done to see whether or not lesion size changed with time or cell type using two-factor (two-way) repeated measure ANOVA, as each lesion/colony was followed over time, and Bonferroni posttest.

#### Mitosis Staining

Ten micromolars of BrdU (Sigma-Aldrich) in cell medium were used to assess mitotic level in the glioma and neuroblastoma cells (Table 2). Immediately after trauma, the medium in every well was replaced with 1000 μl BrdU-laced medium. Cells were maintained as described above. After 24 h, all colonies were fixed for 20 min in 4% formaldehyde (APL).

Blocking and antibody incubations were performed in 10% donkey serum and 0.1% Triton-X in 0.01M PBS. Rinsing was done in 0.01M PBS. All steps were at room temperature unless otherwise stated. The samples were rinsed three times, blocked for 60 min, and then incubated in β-tubulin rabbit antibody (1:500; Covance) overnight at 4°C. The samples were then blocked for 60 min, incubated in Cy2 donkey anti-rabbit (1:50; Jackson ImmunoResearch) for 60 min, and rinsed three times for 10 min. Fixation was performed in 4% formaldehyde for 5 min, followed by rinsing for 10 min three times. DNA was denatured for 5 min with fresh 4M HCL in 0.1% Triton-X in 0.01M PBS. Samples were then rinsed plentifully three times and stored overnight at 4°C in 0.01M PBS. G3G4 mouse anti-BrdU (1:25, DHSB at University of Iowa) incubation was carried out for 60 min followed by rinsing three times. Samples were incubated in Cy3 donkey anti-mouse (1:1000; Jackson ImmunoResearch) for 60 min. The samples were incubated with DAPI (1:1000; Invitrogen) in 0.01M PBS for 3 min, rinsed two times for 5 min. The cover slips were mounted on slides with mowiol 4-88 (Polysciences). Control cultures, not subjected to *in vitro* trauma, were handled in the same way as exposed cultures.

A Nikon E600 flourescence microscope and a Nikon Digital Sight DS-U1 camera (with Nikon NIS Elements software) were used for micrographing. The number of mitotic and non-mitotic cells was assessed using ImageJ64 (NIH). The percentage of mitotic cells was calculated from the number of mitosis and cells. Statistical analyses were done to see whether or not mitosis frequency was different in different cell types or zones surrounding a lesion using two-factor (two-way) ANOVA and Bonferroni posttest. The statistical analysis was done with Prism 5.

### Time Lapse Live Cell Imaging

Cell-IQ (Chipman technologies, Tampere, Finland) was used for integrated incubation, live-cell photography, and image analysis. Controls and flyer-plate exposed colonies of all three cell types were assayed with Cell-IQ (Table 3). In the Cell-IQ experiments, the cells were grown and flyer-plate exposures carried out, as described above. Glioma and Caco-2 were traumatized at a less confluent state than neuroblastoma since these cells tend to aggregate in cell clusters, complicating cell counting, at an earlier (less confluent) stage than neuroblastomas.

Pictures were taken between the 1st and 26th hour posttrauma every 3rd hour. In all three cell types, the lesion periphery is a less cell-dense zone between the cell-free lesion and the confluent cell population. Phase contrast pictures were taken of the lesion periphery – or a confluent area in controls (10× magnification). The area to be photographed was manually chosen so that approximately half of the image area was cell populated. *Z*-stacks of images were used by the Cell-IQ system to produce single all-in-focus images, each of which was cell-counted using the Cell-IQ Analyzer software. Cell count data were normalized in excel: the first time point measurement (cell count) was defined as 100%, and every measurement (cell count) thereafter was a percentage of the first value. The mean, SD of the normalized values, and plots thereof were done with Prism 5.

### Trauma Biomarker – S100B Measurement with Enzyme-Linked Immune-Sorbent Assay

Measurement of calcium-binding protein (S100B) was performed using a commercially available ELISA-kit (Sangtec Molecular Diagnostics AB). A confluent cell layer was subjected to flyer-plate trauma as described above. Repeated insults were performed within minutes (1–3) of the first insult in an adjacent non-traumatized area of confluent cells. Directly after flyer-plate trauma, samples from the cell culture medium were collected. The samples, standards, and controls were added onto a 96-well microplate coated with 2 solid-phase catcher antibodies specific for S100B (Table 4). A S100B-specific detector antibody conjugated with horseradish peroxidase was added. After 2 h incubation at room temperature, the wells were washed, and tetramethylbenzidine (TMB) substrate was added. TMB stop solution was added after 15 min. The absorbance was measured at 450 nm.

### Gene Array

Every glioma sample was harvested from eight colonies on one culture dish. The glioma arrays showed high RNA yield and quality, so such extensive harvesting from eight colonies was not necessary. Sample harvesting was then scaled down. Every neuroblastoma sample was from one colony (Table 5). Twenty-four hours after exposure, all wells were washed with 37°C Hank’s buffered salt solution (HBSS, LifeTechnologies). Two hundred microliters of trizol (LifeTechnologies) were added to each well for 3 min RNA extraction. Cells were scraped off the well bottoms. The trizol–cell suspension was then kept in Eppendorf tubes at −70°C.

Analysis was performed by the Karolinska Institute core facility for Bioinformatics and Expression Analysis (bea.ki.se). After target preparation and hybridization to microarray, RNA was labeled with biotin to produce the final target according to Affymetrix standard procedures (Affymetrix). Labeled cRNA was then hybridized to respective Affymetrix GeneTitan Gene ST 1.1 platform – rat for glioma and human for neuroblastoma. After probing and scanning, all array images were checked in Affymetrix quality control. Following normalization, differential gene expression (up- and downregulation) exposed vs. controls was assessed in terms of fold change in an unpaired *t*-test. Three exposed and three controls of each cell type were included.

Lists of genes that are significantly differentially expressed (*P* ≤ 0.05) were uploaded to the Database for Annotation, Visualization, and Integrated Discovery (DAVID) (david.abcc.ncifcrf.gov) for functional annotation clustering (19).

## Results

**Figure 3:**
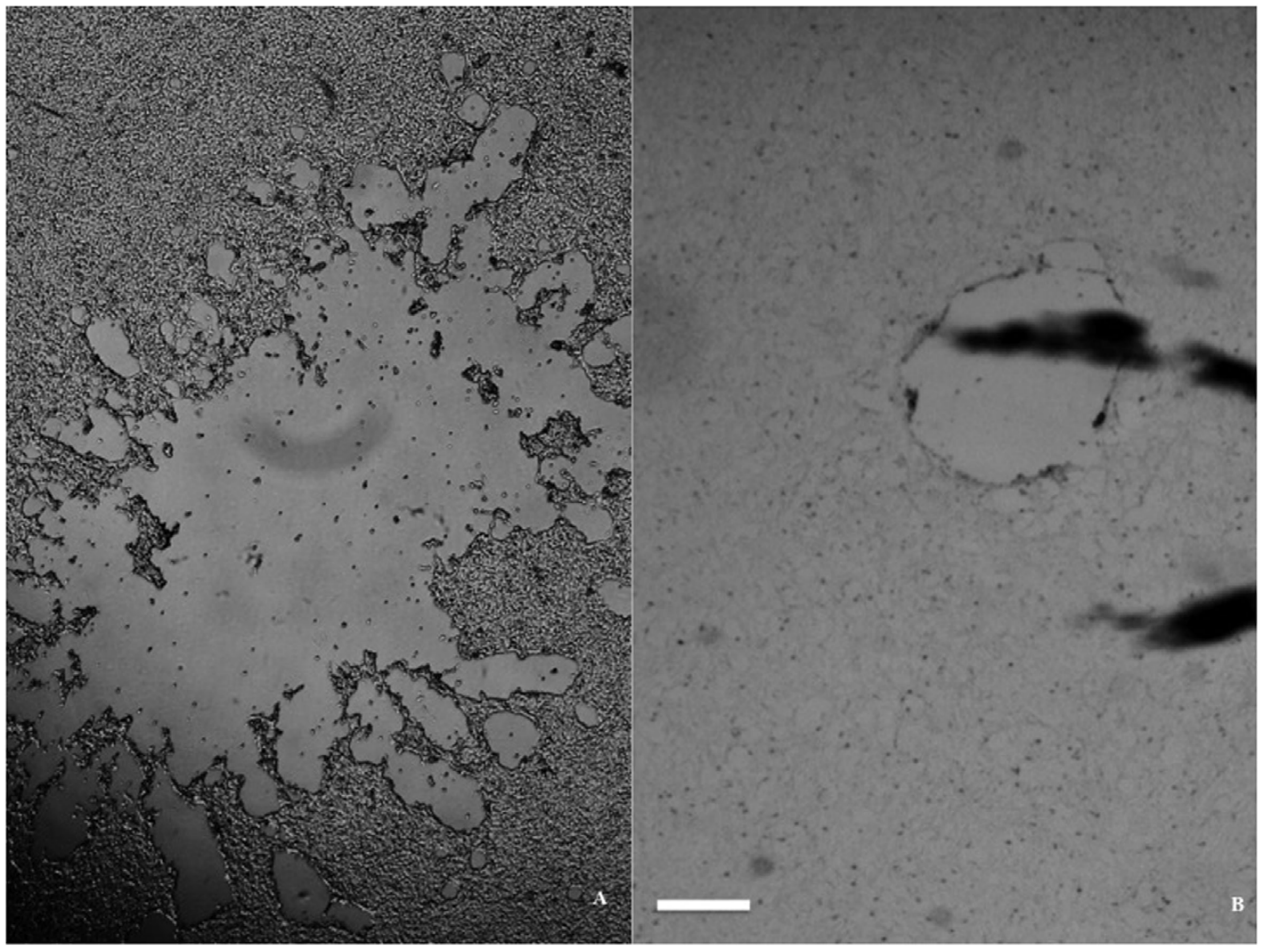
**Lesion size dependence on volume of medium in a glioma monolayer**. **(A)** Large lesion with 0.4 ml medium/well. **(B)** Small lesion with 1 ml medium/well. Both micrographs were taken immediately after trauma exposure, with the scale bar at 1 mm in both pictures.

The impact resulted in a more or less circular injury, surrounded by a reactive zone (the lesion periphery). At a larger distance from the impact, the morphology of the cells appeared fairly intact. The injury zone varied between the different cell lines and was better defined in both glioma and neuroblastoma cultures than in the Caco-2 specimens. In all three cell lines, we observed a bigger lesion when using small medium volume and a smaller lesion upon using a big medium volume (Figure 3, only glioma illustrated). In other words, lesion size changed without exception, as medium volume was changed for the same flyer-plate exposure energy.

### Lesion Size and Morphology

**Figure 4:**
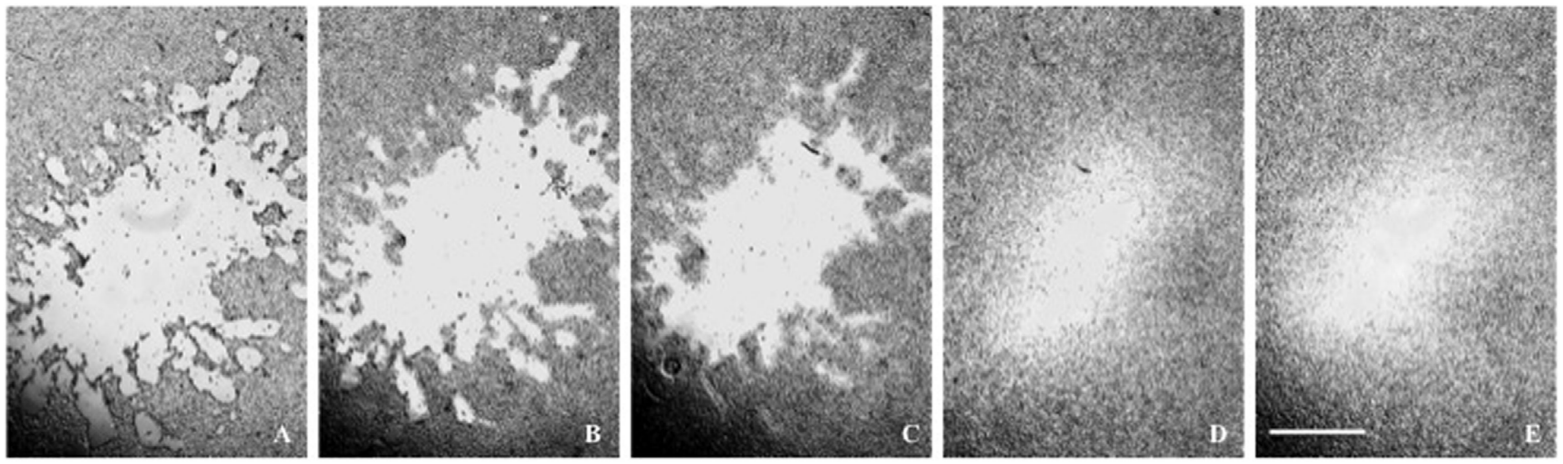
**(A–E)** A glioma lesion posttrauma. The lesion periphery is a less cell-dense zone between the lesion and the confluent cell population. Time posttrauma **(A)** 0.75 h; **(B)** 3.33 h; **(C)** 6.00 h; **(D)** 23.00 h; **(E)** 26 h. The microscope scale is 1 mm for all micrographs.

Live cell cultures were observed and photographed five times post trauma – means (intervals): 0.875 h (0.75–1.00 h), 3.830 h (3.33–4.33 h), 5.835 h (5.50–6.17 h), 23.250 h (23.00–23.50 h), and 26.165 h (26.00–26.33 h). *P* < 0.05 was considered significant. The morphology of the cell types posttrauma was documented with micrographs, an example of which is shown in Figure 4. The micrographs should be free of detached cells and debris since the medium was emptied and fresh medium was added after flyer-plate exposures. Lesions in glioma and neuroblastoma have similar spatial patterns with a whole continuous cell-free zone. Their posttrauma growth characteristics in time are also similar. Caco-2 tends to develop patches of smaller lesions (see Figure 7D). Caco-2 also seems to be less vulnerable to trauma, as some colonies had small or very small central lesions. In all three cell types, the lesion periphery is a less cell-dense zone between the cell-free lesion and the confluent cell population, with reactive and dying cells.

**Figure 5:**
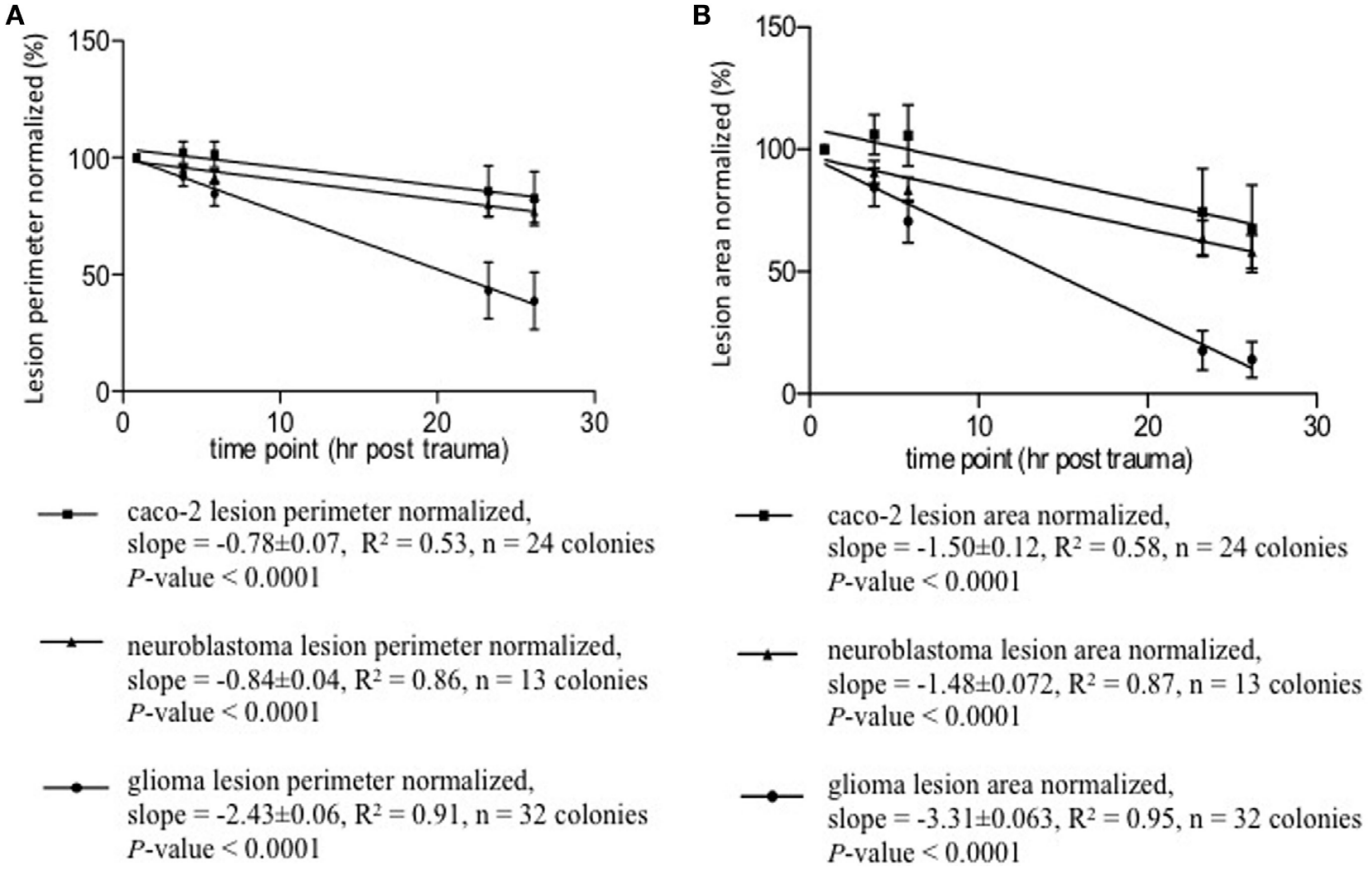
**Lesion size–time relationships appear to be linear, see *r*^2^-values**. A characterizing yet very approximate parameter of regeneration is the slope. Each line represents one cell type with its own slope. The slopes are significantly non-zero, all *P*-values <0.0001. **(A)** Lesion perimeter normalized mean ± SD (%) vs. time posttrauma (hours). **(B)** Lesion area normalized mean ± SD (%) vs. time posttrauma (hours).

The lesion zones were assessed for area and perimeter. Over the observed period, ~0.875–26.165 h, the lesion size in all cell types decreased. Caco-2 colonies tended to have patches of lesions compared to the continuous cell-free zone of the other cell types. For Caco-2, the biggest (chosen by the microscopist) lesion patch in a colony was measured. The lesion size–time relationships are shown in Figure 5. These relationships follow straight lines fairly well, see *r*^2^-values. One characterizing parameter is the slope – each line represents one cell type with its own slope. The slopes are significantly non-zero (all *P* values <0.0001), i.e., lesion size’s change in time is significant and are approximated descriptions of posttrauma regrowth. The neuroblastoma and glioma lesions reduced in size immediately after the trauma – the second observation had a size smaller than the first. The Caco-2 lesions enlarged at the second observation compared to the first. After the second time point, the Caco-2 lesion also decreased in size. The Caco-2 cell cultures initial lesion enlargement followed by size reduction is reflected in its lower *r*^2^-value.

The lesion size–time data were tested for its statistical significance at two levels. First, the overall progression – from the first time point to the last – was tested with two-way repeated measure ANOVA – answering these questions. (1) Does time have a statistically significant impact on the cells’ regeneration post trauma? (2) Does regeneration show statistically significant cell-type specificity? (3) Do the time factor and cell-type factor interact in a statistically significant manner? Then, each observation was compared to the first with Bonferroni test to clarify whether or not any statistically significant change has taken place at each time point relative to the first lesion measurement post trauma.

Two-way repeated ANOVA showed that time accounted for change in lesion perimeter by 34.75% (*P* < 0.0001) and 41.38% (*P* < 0.0001) in lesion area. The time effect within every cell type is significant, i.e., each cell type exhibits significant regeneration over time. Cell type contributed to change in lesion perimeter by 21.74% (*P* < 0.0001) and 22.37% (*P* < 0.0001) in lesion area. The regeneration processes are significantly different between these cell types. The time factor and cell-type specificity also interacted with each other and contributed to change in perimeter by 14.76% (*P* < 0.001) and 10.45% (*P* < 0.001) in lesion area. The differences between cell types regarding regeneration are significantly different over time. That is – the regeneration differences observed among these cell types is not constant over time. The Bonferroni post test was used to analyze the lesion size change at each timepoint compared to the first timepoint after the injury. It showed that regrowth became more pronounced with time. The change in lesion size at most timepoints vs. the first was significant, *P* < 0.05 to *P* < 0.0001. The exceptions were the initial observations (second in neuroblastoma with a change to small to be significant and second and third in Caco-2), where the changes in lesion size had *P* values exceeding 0.05. Lesions in Caco-2 monolayers enlarged initially, whereafter Caco-2 also grew back into the lesion zone. This explains why the lesion size at the third timepoint was not statistically different from the first – the enlarged lesion shrank back in size.

### Time Lapse Live Cell Imaging

**Figure 6:**
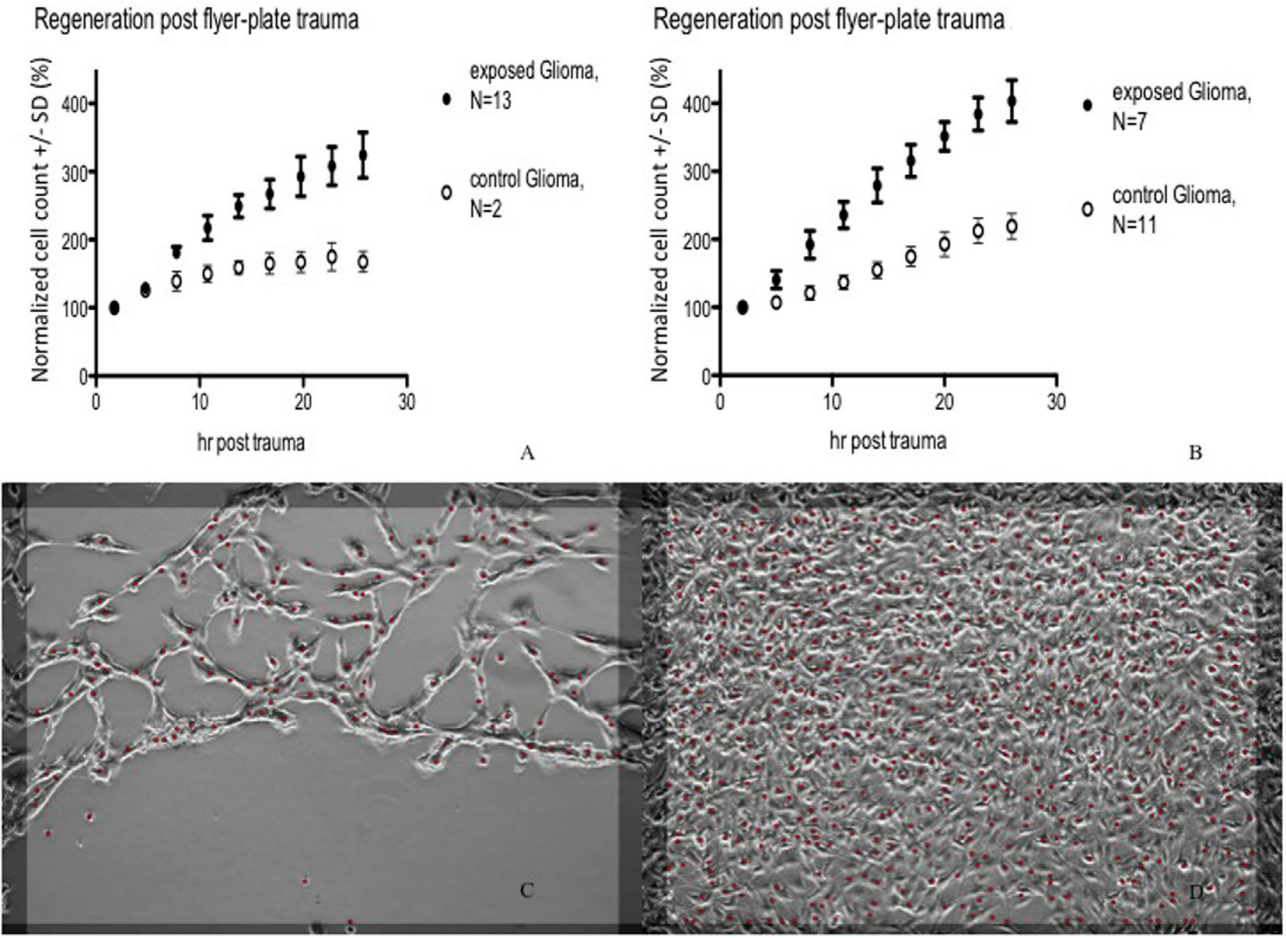
**Cell count (normalized mean ± SD) in lesion peripheries vs. control-colonies of glioma**. Trauma exposures in two different sets of measurements **(A,B)** are shown. Micrographs of live glioma – first **(C)** and last **(D)** images post trauma – illustrating growth at the lesion periphery. The red dots indicate counted cells. Original magnification 10×.

**Figure 7:**
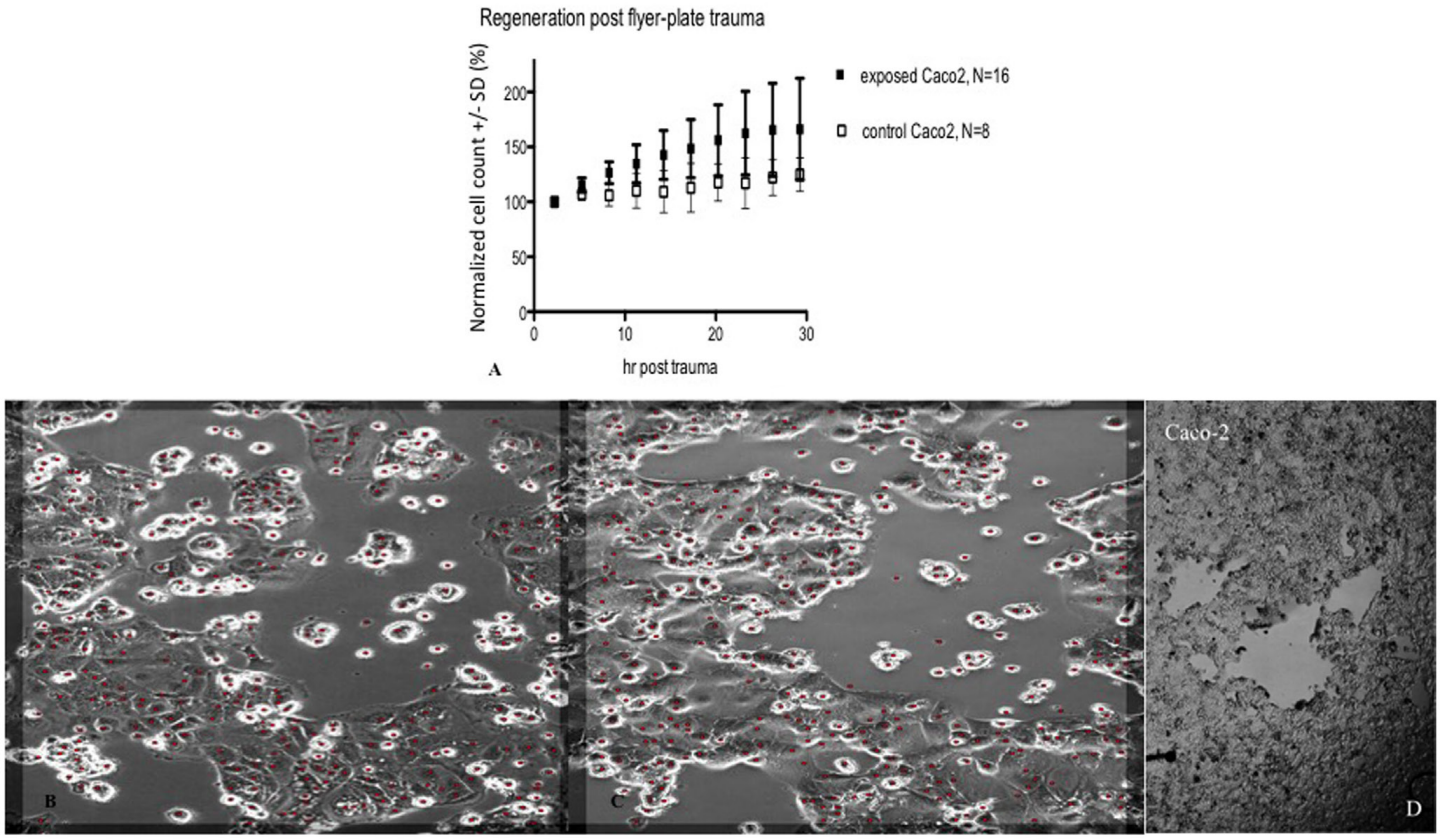
**Cell count (normalized mean ± SD) in lesion peripheries vs. control-colonies of Caco-2, trauma exposures in (A)**. Micrographs of live Caco-2 – first **(B)** and last images post trauma illustrating growth at the lesion periphery **(C)**. The red dots indicate counted cells. Original magnification 10×. Incubated, photographed, and counted by Cell-IQ. **(D)** shows microphotograph overview of injury to Caco-2 cells that typically resulted in multiple separated patches devoid of cells.

**Figure 8:**
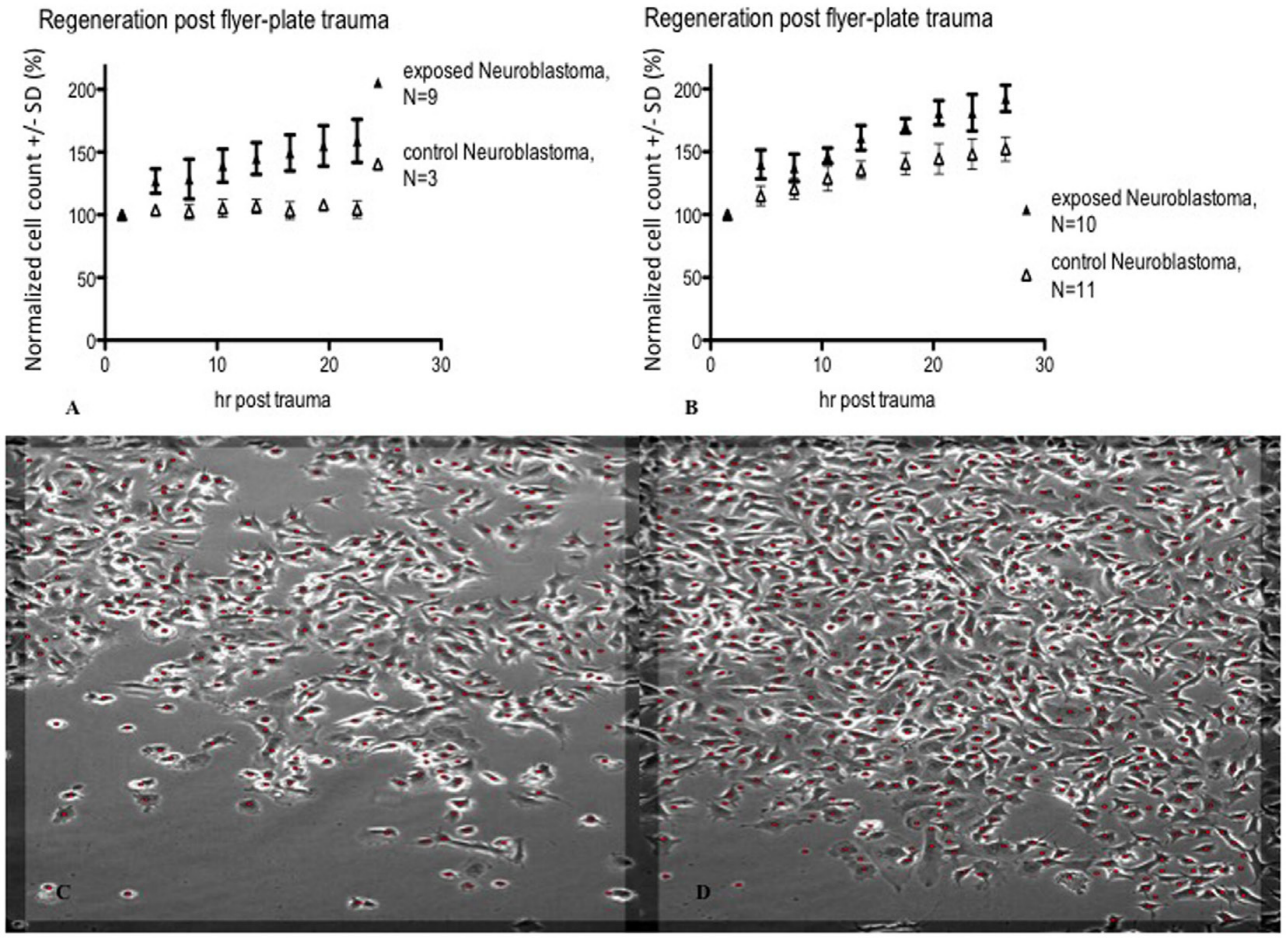
**Cell count (normalized mean ± SD) in lesion peripheries vs. control-colonies of neuroblastoma**. Trauma exposures in two different sets of measurements **(A,B)** are shown. Micrographs of live neuroblastoma – first **(C)** and last **(D)** images post trauma – illustrating growth at the lesion periphery. The red dots indicate counted cells. Original magnification 10×. Incubated, photographed, and counted by Cell-IQ.

In subsequent experiments, Cell-IQ was used to achieve a more dynamic description of the cell populations after the trauma. With this instrument, the cell cultures can be analyzed in real time without removing them from the incubator environment. Cell-IQ experiments were conducted once on Caco-2 and twice on glioma and neuroblastoma. Cell growth in the lesion periphery was observed in all cell types (Figures 6–8). Normalized cell counts in lesion peripheries of traumatized colonies were compared to non-exposed colonies. The biggest difference between exposed and controls was observed in glioma followed by neuroblastoma, while the difference was small in Caco-2. In Caco-2, the difference may be less obvious due to the bigger variation between colonies, yet the means of the exposed and controls still follow two separate lines. The smaller difference in Caco-2 may also be attributed to Caco-2 cells growing at a slower rate than the other cell types. From seeding to confluence, it takes roughly 2 days for Caco-2 and 1 day for glioma and neuroblastoma. The difference could be more evident in Caco-2 with a longer observation period, here circa 1st to 26th hour post trauma. The sizable intercolony variation is also caused by Caco-2’s tendency to clump and grow atop one another – making cell counting difficult. Finally, Caco-2 does exhibit more intercolony variations than the other cell types in terms of sizes and forms of lesions. The same kinds of lesion formation were observed here as described earlier in the photomicroscopy experiments – continuous lesions in glioma and neuroblastoma, and patched lesions in Caco-2.

### Mitosis

**Figure 9:**
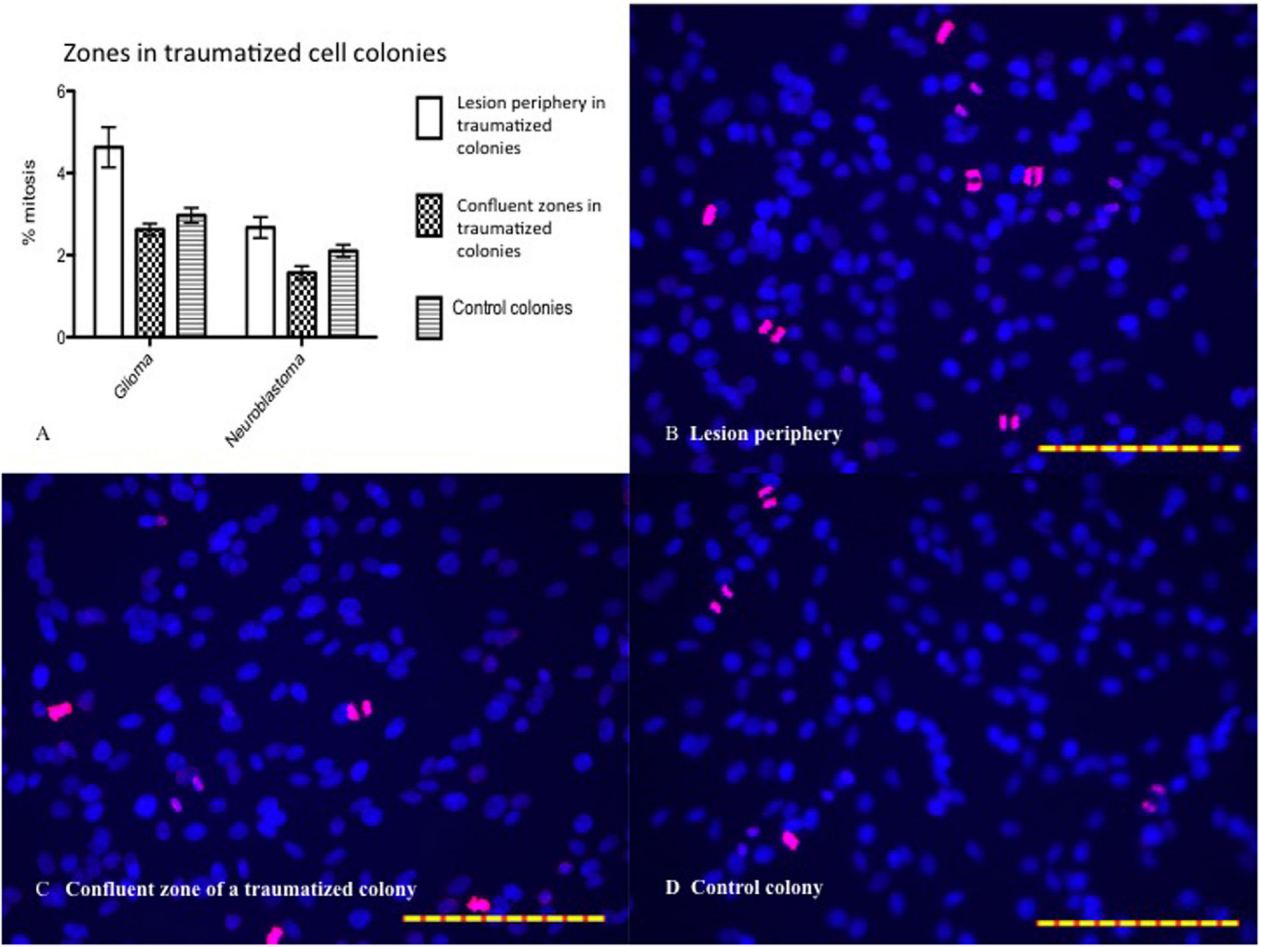
**(A)** Mitosis percentage statistically analyzed by two-way ANOVA and Bonferroni posttest. Mitosis percentage is significantly different between cell types (*P* < 0.0001). The difference between periphery and confluent zones is significant in glioma (*P* < 0.0001) and neuroblastoma (*P* < 0.01). The difference between periphery and control is significant in glioma (*P* < 0.0001), but not in neuroblastoma (*P* > 0.05). The confluent zones and controls are mitotically similar, in both cell types (*P* > 0.05). Mean ± SEM. *n* glioma periphery = 13, *n* glioma confluent = 17, *n* glioma control = 19; *n* neuroblastoma periphery = 20, *n* neuroblastoma confluent = 18, *n* neuroblastoma control = 17. **(B)** BrdU (pink) and DAPI (blue) stained glioma (24 h posttrauma) with high percentage of mitosis in the periphery. **(C)** Low percentage of mitosis in the confluent zone. **(D)** Low percentage of mitosis in controls. The scales in all images are 1 μm.

BrdU labeling was employed in order to reveal whether an increased frequency of mitosis might contribute to the decrease in lesion size over time. BrdU and DAPI staining revealed zonal differences in mitosis between the highest percentage in the lesion periphery and the lowest in the confluent zone, for both cell types (Figure 9). Only images of glioma are shown, as those of neuroblastoma are similar. Two-way ANOVA and Bonferroni post test showed that mitosis percentage is significantly different between cell types (*P* < 0.0001). The difference in periphery vs. confluent zones is significant in glioma (*P* < 0.0001) and neuroblastoma (*P* < 0.01). The difference between the periphery and confluent zones/controls is significant in glioma (*P* < 0.0001), but not in neuroblastoma (*P* > 0.05). The confluent zones and controls are mitotically similar, in both cell types (*P* > 0.05).

### S100B

**Figure 10:**
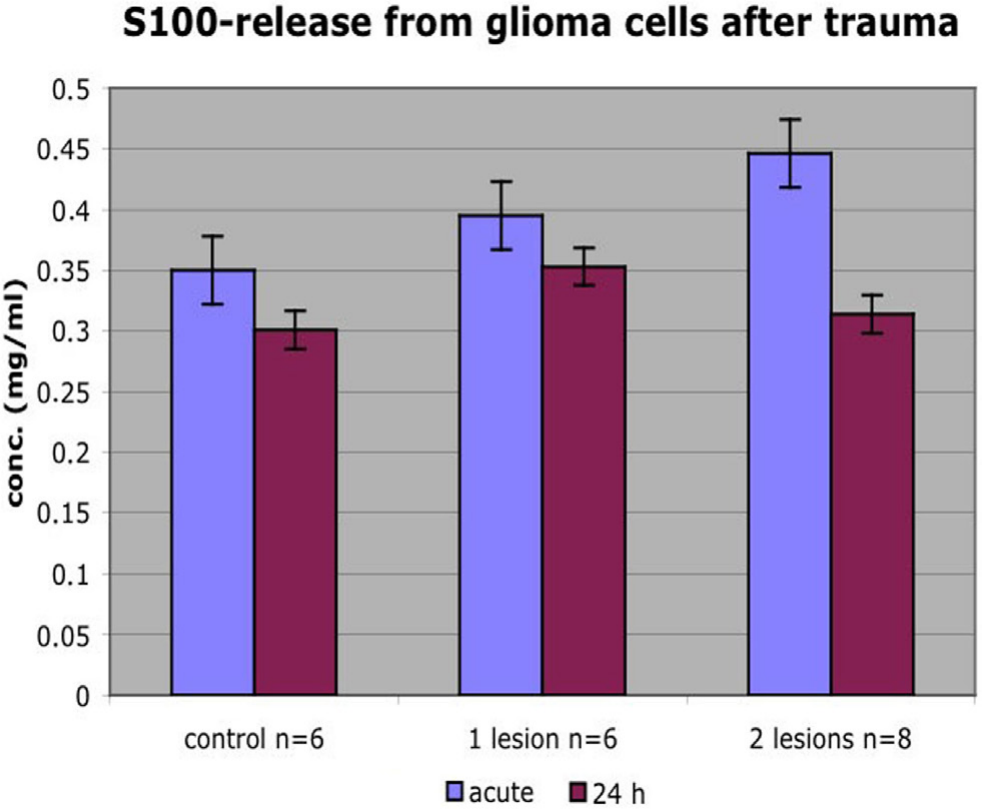
**Colonies that received more insults had higher S100B concentrations**. S100B concentration was lower 24 h posttrauma than immediately after trauma. Mean ± SEM.

The calcium-binding protein S100B is considered to be a sensitive biomarker for cellular injury. The content of S100B was analyzed in the medium in order to reveal if cells that were detached within the impact zone were injured or just detached. Due to the relatively high levels of S100B in non-traumatized colonies, repeated trauma was performed. We showed that colonies with higher number of insults had higher S100B concentrations. S100B concentration was lower 24 h post trauma than immediately after trauma. Thus, S100B showed an acute and graded increase in response to the impact (Figure 10).

### Gene Array

**Figure 11:**
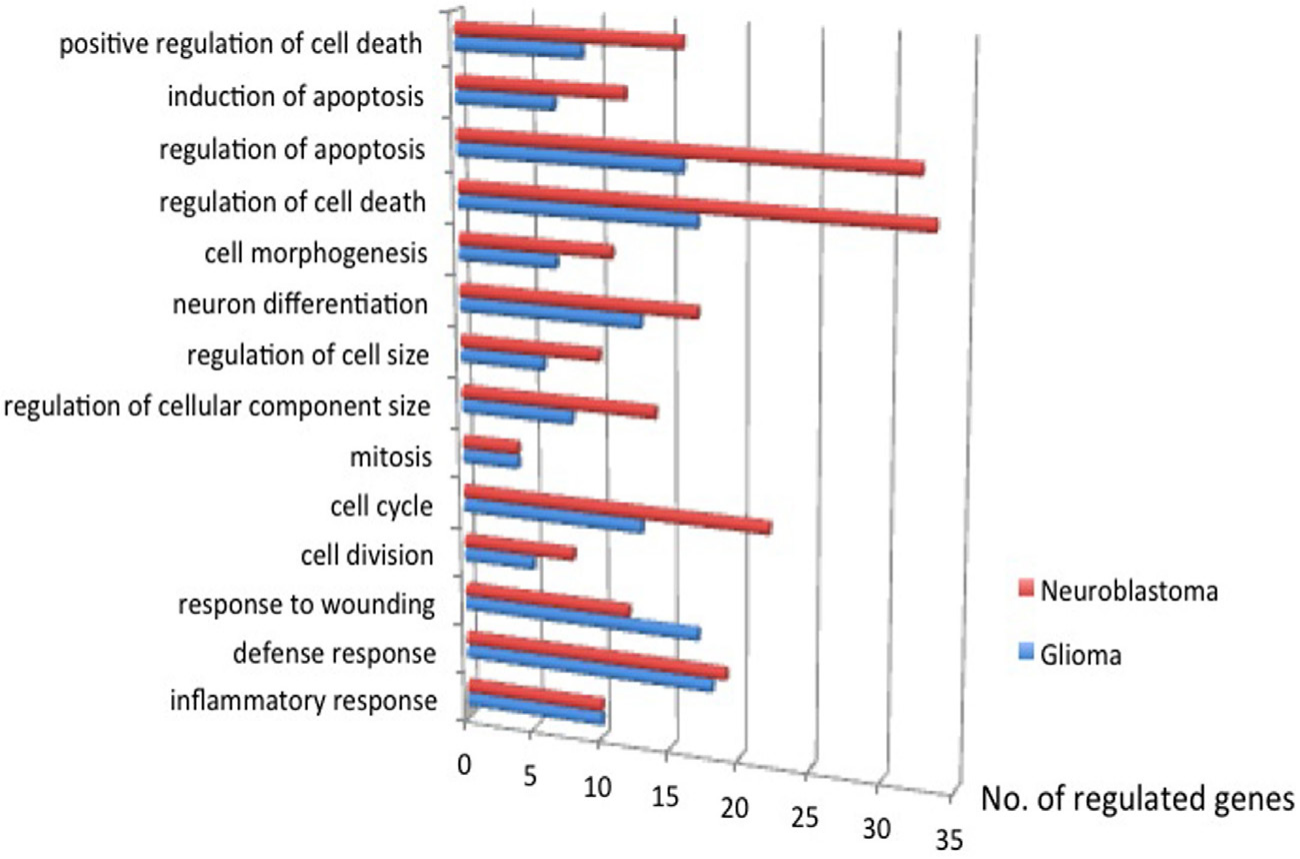
**The number of genes regulated, traumatized vs. control, *P* ≤ 0.05**. Immune response, cell cycle/division, and cell death are three themes of regulated gene expression.

Gene expression microarrays in both glioma and neuroblastoma cultures were used to identify gene families with significant shifts in expression after the injury. The DAVID database gene ontology toolbox was used to reveal clusters of reactive genes and to find out if there were any cell-type specific patterns. Immune response, cell cycle/division, and cell death are three themes of regulated (both up and downregulations) gene expression when comparing traumatized vs. control cells. The DAVID database annotates gene groups with terms (names). The number of regulated genes in each term is shown in Figure 11. These gene groups’ expression was regulated in both cell types, though not considered highly regulated (enrichment score <1.3). Related terms are grouped as clusters (Tables 6 and 7). Each cluster is ranked relative to all gene clusters in a study with an enrichment score. The higher the enrichment score, the more regulated a cluster is. An enrichment score is based on the mean of *P* values (of the terms in that cluster). Enrichment score of 1.3 is equal to −log *P*-value of 0.05 (19). Table 6 (glioma) and Table 7 (neuroblastoma) list the clusters above the enrichment score cut-off at 1.3. In both analyzed cell types, genes related to membrane functions appeared highly regulated. In the neuroblastoma cells, an interesting pattern in the regulation of gene expression for steroid hormones was observed.

**Table 6:** **Regulated genes (traumatized vs. control) in glioma, *P* ≤ 0.05**.

Annotation cluster 1, enrichment score: 1.79	Term	*P*-value
GOTERM_BP_FAT	GO:0007186 ~ G-protein coupled receptor protein signaling pathway	0.0005
GOTERM_BP_FAT	GO:0007166 ~ cell surface receptor linked signal transduction	0.0028
GOTERM_BP_FAT	GO:0050877 ~ neurological system process	0.0078
GOTERM_BP_FAT	GO:0009593 ~ detection of chemical stimulus	0.0137
GOTERM_BP_FAT	GO:0007606 ~ sensory perception of chemical stimulus	0.0138
GOTERM_BP_FAT	GO:0051606 ~ detection of stimulus	0.0141
GOTERM_MF_FAT	GO:0004984 ~ olfactory receptor activity	0.0180
GOTERM_BP_FAT	GO:0007600 ~ sensory perception	0.0187
GOTERM_BP_FAT	GO:0050906 ~ detection of stimulus involved in sensory perception	0.0231
GOTERM_BP_FAT	GO:0050911 ~ detection of chemical stimulus involved in sensory perception of smell	0.0305
GOTERM_BP_FAT	GO:0007608 ~ sensory perception of smell	0.0313
GOTERM_BP_FAT	GO:0050907 ~ detection of chemical stimulus involved in sensory perception	0.0344
GOTERM_BP_FAT	GO:0050890 ~ cognition	0.0386

*Top functional annotation cluster, enrichment score >1.3*.

**Table 7:** **Regulated genes (traumatized vs. control) in neuroblastoma, *P* ≤ 0.05**.

	Term	*P*-value
**Annotation cluster 1, enrichment score: 1.81**		
GOTERM_CC_FAT	GO:0031974 ~ membrane-enclosed lumen	0.0021
GOTERM_CC_FAT	GO:0043233 ~ organelle lumen	0.0056
GOTERM_CC_FAT	GO:0070013 ~ intracellular organelle lumen	0.0065
GOTERM_CC_FAT	GO:0005654 ~ nucleoplasm	0.0132
GOTERM_CC_FAT	GO:0031981 ~ nuclear lumen	0.0230
GOTERM_CC_FAT	GO:0044451 ~ nucleoplasm part	0.0501
GOTERM_CC_FAT	GO:0005730 ~ nucleolus	0.1888
**Annotation cluster 2, enrichment score: 1.78**		
GOTERM_BP_FAT	GO:0030521 ~ androgen receptor signaling pathway	0.0020
GOTERM_MF_FAT	GO:0035257 ~ nuclear hormone receptor binding	0.0024
GOTERM_MF_FAT	GO:0050681 ~ androgen receptor binding	0.0025
GOTERM_MF_FAT	GO:0035258 ~ steroid hormone receptor binding	0.0027
GOTERM_BP_FAT	GO:0030518 ~ steroid hormone receptor signaling pathway	0.0056
GOTERM_MF_FAT	GO:0051427 ~ hormone receptor binding	0.0058
GOTERM_BP_FAT	GO:0030522 ~ intracellular receptor-mediated signaling pathway	0.0216
GOTERM_MF_FAT	GO:0003713 ~ transcription coactivator activity	0.2873
GOTERM_MF_FAT	GO:0008134 ~ transcription factor binding	0.4615
GOTERM_MF_FAT	GO:0003712 ~ transcription cofactor activity	0.6143
**Annotation cluster 3, enrichment score: 1.42**		
INTERPRO	IPR013787:S100/CaBP-9k-type, calcium binding, subdomain	0.0149
INTERPRO	IPR001751:S100/CaBP-9k-type, calcium binding	0.0169
UP_SEQ_FEATURE	calcium-binding region:1; low affinity	0.0255
PIR_SUPERFAMILY	PIRSF002353:S-100 protein	0.0317
UP_SEQ_FEATURE	calcium-binding region:2; high affinity	0.0384
SP_PIR_KEYWORDS	EF hand	0.3661
**Annotation cluster 4, enrichment score: 1.32**		
GOTERM_BP_FAT	GO:0000723 ~ telomere maintenance	0.0193
GOTERM_BP_FAT	GO:0032200 ~ telomere organization	0.0217
GOTERM_MF_FAT	GO:0008094 ~ DNA-dependent ATPase activity	0.0628
GOTERM_BP_FAT	GO:0060249 ~ anatomical structure homeostasis	0.2044
**Annotation cluster 5, enrichment score: 1.26**		
GOTERM_BP_FAT	GO:0010921 ~ regulation of phosphatase activity	0.0278
GOTERM_BP_FAT	GO:0035303 ~ regulation of dephosphorylation	0.0712
GOTERM_BP_FAT	GO:0043666 ~ regulation of phosphoprotein phosphatase activity	0.0827
**Annotation cluster 6, enrichment score: 1.26**		
GOTERM_CC_FAT	GO:0005740 ~ mitochondrial envelope	0.0105
GOTERM_CC_FAT	GO:0044429 ~ mitochondrial part	0.0175
GOTERM_CC_FAT	GO:0031966 ~ mitochondrial membrane	0.0176
GOTERM_CC_FAT	GO:0031967 ~ organelle envelope	0.0183
GOTERM_CC_FAT	GO:0031975 ~ envelope	0.0189
GOTERM_CC_FAT	GO:0005743 ~ mitochondrial inner membrane	0.0934
GOTERM_CC_FAT	GO:0031090 ~ organelle membrane	0.1016
GOTERM_CC_FAT	GO:0019866 ~ organelle inner membrane	0.1465
SP_PIR_KEYWORDS	mitochondrion inner membrane	0.1580
SP_PIR_KEYWORDS	Mitochondrion	0.1866
GOTERM_CC_FAT	GO:0005739 ~ mitochondrion	0.3041

*Top functional annotation clusters, enrichment score >1.3*.

## Discussion

A reliable, highly reproducible *in vitro* trauma model could have various implications in the study of cellular responses to trauma. Multiple *in vitro* trauma models, including *in vitro* transection, compression, barotrauma, acceleration/deceleration, hydrodynamic models, cell stretch, and three-dimensional trauma, have previously been described (20, 21). In this article, we describe a novel *in vitro* trauma model, such as the flyer-plate model, that provides the possibility to investigate high-energy trauma with cavitation on the cellular level. In this study, we evaluated if this method can generate reproducible results with the three tested cell lines. Cell lines were chosen over primary cell cultures due to their robust growth and easy maintenance, and thereby their relative stability over time and the possibility to perform high throughput studies. By measuring changes in lesion size, cell division, release of cell injury markers, changes in gene activation, and by live cell imaging post trauma, we evaluated this model as a tool for *in vitro* trauma studies. The results imply that the flyer-plate model can generate robust and reproducible data with such cell lines. By selecting cell lines of neuronal origin, the model gives potential insight in transient mechanisms in high-energy trauma, such as cavitatiton, in CNS trauma. Such studies can hopefully be of relevance for the understanding of components in TBI that would not be accessible with *in vivo* methods. The system used herein, with a single monolayer on a rigid surface, is a simplification of whole tissue and even more so of whole organs and the responses on trauma within this system should therefore be interpreted with great care.

The flyer-plate has a number of characteristics resembling high-energy trauma. The insult delivered is momentary, compared to prolonged time of exposure, as in some previously described models (21). Also, the model enables studies of cellular behavior in colonies rather than in single cell trauma systems such as microtransection (20, 21) enabling high throughput studies by averaging responses in multiple, traumatized cells at a time. In our system, the energy is easily adjusted and controlled by adjusting laser energy and medium volume, and it is possible to measure the energy levels at the site of delivery (between 500 and 619 mJ recorded in the current study) and to photograph the insult in the well in real time (14). In addition, the injury and the observed post trauma growth exhibited good reproducibility.

Medium volume per well can be varied and affects the magnitudes of force, and therefore trauma, in our flyer-plate model (14, 18). Cavitation bubble formation is also dependent on the medium volume. At larger volumes of medium, cavitation bubble formation rarely occurs and thus the resulting trauma is less pronounced. On the other hand, small volumes cause widespread trauma to the cell monolayer. Four hundred microliters of visibly bubble-free medium per well were chosen for this study because that volume induced an injury of reasonable size to be easily documented and easily reproduced. In agreement with Sondén et al., we observed surface and bottom cavitation using small medium volume, while cavitation only occurred at well bottom when using big medium volume. Also observed were the larger lesions in wells with smaller medium volumes. Surface cavitation was thought to have caused the difference in lesion size (14, 18). All of the cell types studied with the flyer-plate setup were affected by the cavitation. We made the same observation in all our studied cell types. Cavitation seems to exert injurious effects on all cell types studied with the flyer-plate setup.

The trauma applied in the model will remove cells from the central area of the lesion. Thereafter dividing cells will regrow into this area by migration, or by growth of neurites, enabling studies of cell migration and neurite growth after trauma. Potentially, the speed and pattern of migration after trauma can be studied in a system like this, drugs affecting growth or adhesion, can be added and their effect evaluated. To study the basic growth pattern in our system, the lesion area and the lesion perimeter were studied in three different cell types: neuroblastoma, Caco-2, and glioma. A description of this posttrauma growth is shown in Figure 5 where the lesion size–time relationships are plotted. Analysis with ANOVA showed significant change of lesion size in time, cell-type specificity, and time–cell type interaction. As seen in Figure 5, glioma cells had the fastest regrowth pattern, both when measured with changes in perimeter and lesion area. Caco-2 differed from the other two cell types and besides a central lesion the trauma resulted in patches devoid of cells distant from the central lesion. The reason for this is unclear, but it is potentially interesting since Caco-2 shares features with BBB such as tight junctions that might affect the lesion size after trauma.

With use of an automated and integrated incubating, imaging, and analysis system, Cell-IQ (22, 23), live cultures were studied. This system enabled a number of cell events, such as cell number, cell death, migration, shape, size, morphology, and recognition of cell type to be analyzed in an automated way. In all cell types, growth after *in vitro* trauma, determined by measuring cell number, was more pronounced in the exposed colonies than the controls (as shown in Figures 6–8) in measuring cell number. The Cell-IQ data showed the same trends as lesion size–time relationships – all cell types exhibited time-dependent growth during the first day post trauma and each cell type also seemed to grow in specific patterns. It is important to keep in mind that the exposed area is defined as the central area that is devoid of cells directly after trauma and therefore likely attracts more migrating cells than the already confluent area outside the central lesion area. This is likely in part an explanation behind the pronounced differences in growth in exposed cultures compared with controls. Whether or not the seemingly increased cell growth after exposure is a matter of stimulation induced by the trauma or not needs further investigation. Nonetheless, we could show cell growth by our method of counting cell numbers, enabling future studies including different aspects of treatment, change of growth substrate, etc.

To further investigate the cellular response to *in vitro* trauma, mitosis was studied by the use of BrdU–DAPI stainings. The number of mitosis was calculated in the zone just bordering the central area devoid of cells, called the lesion periphery, and in the confluent zone outside the lesion periphery, see Figure 9. The mitotic activity in the lesion periphery was shown to be higher than that in the confluent zone in traumatized colonies, and this trend was seen in both gliomas and neuroblastomas but with a more pronounced pattern in glioma cells. The mitotic level in glioma lesion periphery was higher than that in controls – a difference not seen in neuroblastoma, perhaps due to neuroblastoma’s lower growth rate. In both cell types, no differences were seen between the confluent zones and controls with regard to mitosis (Figure 9). Since the border zone is a mixture of injured but viable cells, normal cells, and dying cells, it is less likely that the mitosis observed can be attributed only to cells migrating into the zone after injury. It seems, thus, that the trauma induces a mitotic activity in the border zone and that this increase in mitotic activity can be easily measured.

Yet, another method to describe the injury and the cells’ response to injury is measuring the release of injury biomarkers. One such potential marker is the calcium-binding protein S100B, suggested to be a serum marker in TBI and therefore of interest as a marker for cell injury in our system. We can show that cell culture medium in colonies that received a higher number of insults had higher S100B concentration. The lower S100B concentration 24 h posttrauma probably reflects the amount of cell lesion/dying cell directly after trauma vs. fewer viable but S100B releasing cells at a later timepoint. Furthermore, S100B released immediately after injury had probably degraded (S100B’s half-life is about 0.5–2 h) or been reabsorbed by surviving cells at 24 h contributing to lower concentrations.

To get an overview of biological events after flyer-plate trauma, microarrays were performed on material collected from flyer-plate traumatized cultures. We performed a gene ontology-based analysis and searched for trends rather than individual gene expression changes. The material was analyzed regarding regulation of different gene families. Microarray analysis at 24 h posttrauma revealed regulations in important cell functions, serving many therapeutic targets. In glioma, the top functions included ion-channel receptors, G-protein-coupled receptors (GPCRs, a large family of cell membrane receptors involved in a wide variety of cell signaling processes), and enzyme-linked receptors. In neuroblastoma, the top regulated functions were membrane-enclosed organelle, steroid receptor binding, calcium binding, telomere maintenance, phosphorylation regulation, and mitochondria. In both cell types, differential gene expression was seen in immune response, cell cycle, and cell death. With the help of microarray analysis 24 h posttrauma, we obtained an idea of which functions were regulated, and therefore potentially how trauma, as applied in our method, could influence regulation in the studied cell types. One example is G-proteins. GPCRs are encoded by 1–3% of the genes in our genome. This superfamily of receptors serves as targets for many drugs (24, 25). G-protein activation triggers a number of downstream cellular events (26). Cell surface receptors include ion-channel receptors, GPCRs, and enzyme-linked receptors. A magnitude of cellular processes and eventually transcription are elicited with their activation (26). Ion-channel receptors (also called ligand-gated ion channels) are frequently found in neurons. Most enzyme-linked receptors are protein kinases or protein kinase associated. There are 518 identified protein kinases, about 1.7% the human genome. Many cellular processes are results of phosphorylation by protein kinases: metabolism, transcription, cell cycle progression, cytoskeletal rearrangement, cell movement, apoptosis, and differentiation. Phosphorylation is also important in homeostasis, functions of the nervous and immune systems, and intercellular communication during development (27). Cell surface receptors – such as glutamate receptors, purinergic receptors, and *N*-methyl-d-aspartate receptors (NMDAR) – affect neurological processes and are involved in TBI responses (28).

Identification of cell surface receptors (including GPCR) as the top families of regulated genes could be of special interest since their downstream actions are seen in response to trauma and as such could be interesting future targets of intervention. The top regulated gene clusters of neuroblastoma deal with membrane-enclosed organelle, steroid receptor binding, calcium binding, telomere maintenance, phosphorylation regulation, and mitochondria. Organelles’ formation takes place during cell divisions. That organelle expression is among the top regulated functions seems reasonable as cell division, cell cycle, mitosis, regulation of cellular component size, regulation of cell size, neuron differentiation, and cell morphogenesis are regulated in neuroblastoma (Figure 10). Some steroid receptors and steroids (e.g., testosterone, dihydrotestosterone, and estrogen) have been reported to have preclinical neuroprotective effects (29). However, some studies also attribute cell-death induction to steroid signaling (30). Steroid signaling’s differential expressions might provide relevant information to these discussions.

Following TBI, cellular calcium influxes can lead to enzyme activation and mitochondrial Ca^2+^ overload causing neuronal damage. Tubulin, microtubule-associated proteins, neurofilaments, glutamate receptors, membrane transporters, cell-adhesion molecules, protein kinases, and phosphatases are examples of calpain substrates. Calpains are, in turn, proteases that become activated at high concentrations of Ca^2+^. Ca^2+^ homeostasis is necessary for many functions of the nervous system: growth, development, neurotransmission, and distinct patterns of differential gene expression in neurons. Perturbed calcium-mediated pathways in neurons may cause various pathophysiologies (28, 31, 32). The telomere and telomere-associated proteins (e.g., telomerase) regulate the neuronal response to oxidative stress and DNA damage associated with TBI. These proteins may be important for neuronal viability. Some studies suggest that telomerase could be antiapoptotic at neuronal insults and preserve mitochondrial DNA integrity and mitochondrial membrane potential (33).

Disturbed calcium homeostasis upon TBI may result in the activation of kinases and phosphatases. This causes changed levels of phosphorylation (by kinases) and dephosphorylation (by phosphatases). Dephosphorylated neurofilaments are, for instance, a result of such events (34). Mitochondria become dysfunctional post-TBI through mechanisms, such as calcium influx into mitochondria, mitochondrial swelling, the release of reactive oxygen species (ROS), and altered mitochondrial membrane potential. Energy metabolism as a result becomes altered (28, 31, 32). In previous studies, we have performed similar gene ontology analysis in three different TBI models (4). One major difference between such *in vivo* models and the current sets of data is the lack of invading inflammatory cells in the flyer-plate model. Thus, not surprisingly, we see a limited inflammatory response in the gene ontology analysis of the current material, whereas we have an obvious metabolic load and changes in families related to membrane functions.

### Limitations and Advantages

It is important to bear in mind that no *in vitro* trauma model can mimic the *in vivo* situation and that the use of a single cell system is a simplification of whole tissue. On the other hand, confounding factors found *in vivo* can be eliminated in an *in vitro* model, potentially elucidating underlying mechanisms in neurotrauma. Furthermore, *in vitro* models can be used as a first model system in evaluating different therapeutic interventions. Compared to other models described (20), our model produces high-energy cavitation trauma, which is, to the best of our knowledge, unique. One limitation with the present study is the lesion size and morphological assessment of the different injury zones. We chose to have one observer doing all of the observations and defining the different zones between the different observations/measurements to make the observations as consistent as possible. The areas devoid of cells and the areas with confluent layers of cells are obviously easy to define but the area in between these, the lesion border, showed a bigger morphological variability regarding cell density, the patterns of the scattered, injured cells and cell morphology. Nonetheless, this area from a morphological standpoint was clearly different from the other two areas, and we therefore chose to make the calculations present in this study based on presence of this zone.

The trauma in our model can be graded by changing medium volume that enables us to study, in more detail, the biomechanics on a single cell level. Such studies could be of importance for the study of strain and strain rate in cell cultures. The possibility to grade the trauma also allows for future studies investigating thresholds in trauma and their characteristics in more detail because microarrays at different levels of trauma could be performed with relative ease. For example, trauma could be incited with or without cavitation to identify changes in gene expression patterns, and thereby indicating genes of special interest. Another potential study could include environmental changes, such as the effects of hypoxic conditions.

We conclude that the flyer-plate model could be a reliable platform for more detailed studies on the regulation of selective biological functions after high-energy trauma, with or without cavitation.

## Author Contributions

YC: performed the study, data analysis, and wrote the manuscript. EM: participated in most of the experiments. AS: provided expert knowledge on flyer-plate. MS: supervised the project and drafting. MB: performed the S100B study. MR: contributed to conception, design, and supervision. All authors read and approved the final manuscript.

## Conflict of Interest Statement

The authors declare that the research was conducted in the absence of any commercial or financial relationships that could be construed as a potential conflict of interest.
